# Projected savings through public health voluntary licences of HIV drugs negotiated by the Medicines Patent Pool (MPP)

**DOI:** 10.1371/journal.pone.0177770

**Published:** 2017-05-25

**Authors:** Sandeep Juneja, Aastha Gupta, Suerie Moon, Stephen Resch

**Affiliations:** 1 Medicines Patent Pool, Geneva, Switzerland; 2 Harvard T.H. Chan School of Public Health, Boston, United States of America; UNAIDS, GUYANA

## Abstract

The Medicines Patent Pool (MPP) was established in 2010 to ensure timely access to low-cost generic versions of patented antiretroviral (ARV) medicines in low- and middle-income countries (LMICs) through the negotiation of voluntary licences with patent holders. While robust data on the savings generated by MPP and other major global public health initiatives is important, it is also difficult to quantify. In this study, we estimate the savings generated by licences negotiated by the MPP for ARV medicines to treat HIV/AIDS in LMICs for the period 2010–2028 and generate a cost-benefit ratio–based on people living with HIV (PLHIVs) in any new countries which gain access to an ARV due to MPP licences and the price differential between originator’s tiered price and generics price, within the period where that ARV is patented. We found that the direct savings generated by the MPP are estimated to be USD 2.3 billion (net present value) by 2028, representing an estimated cost-benefit ratio of 1:43, which means for every USD 1 spent on MPP, the global public health community saves USD 43. The saving of USD 2.3 billion is equivalent to more than 24 million PLHIV receiving first-line ART in LMICs for 1 year at average prices today.

## Introduction

Improving access to medicines in low- and middle-income countries (LMICs) has been a central focus of global health efforts over the past two decades. Apart from community mobilisation and competition law challenges, many global initiatives that aim to broaden access to health technologies in LMICs and thereby improve public health have been created, including large-scale donor funds such as the Gavi Alliance, Global Fund to Fight AIDS, Tuberculosis and Malaria, UNITAID to shape global medicines markets for public health, advance market commitments (eg, for pneumococcal vaccines), a global subsidy (for antimalarial drugs), the priority review voucher (for innovative medicines for neglected diseases), pooled procurement for ARVs, and voluntary licensing mechanisms such as the Medicines Patent Pool (MPP), among others.

Many of these initiatives stem from the global movement to improve access to HIV treatment, which had reached 18.2 million people by 2016. [1,2] Despite major progress, the HIV pandemic continues to pose new challenges, particularly regarding how to ensure ongoing access to quality-assured, affordable medicines in the countries hardest hit. Access to new and existing ARV medicines is more crucial than ever, with the UNAIDS Fast Track targets [3,4] aiming for rapid scale-up of the number of people on antiretroviral therapy (ART) to reach approximately 30 million people by 2020.

Generic competition and supply have played a critical role in reducing prices and improving affordability. Waning et al (2009) [5] demonstrated that originator tiered prices for ARVs in LMICs were 23–498% higher than their generic equivalents. In general, when there is a large volume of demand (as with ARVs), generic competition has proven to be more reliable and sustainable than voluntary price discounts (i.e. tiered pricing) for reducing prices [6]. However, ensuring that newer ARVs are available as low-priced generics requires addressing increased patenting in LMICs. Several strategies are available to do so, with one of them being voluntary licensing [7].

The MPP was established in 2010 to facilitate timely access to low-cost generic versions of patented ARV medicines in LMICs through the negotiation of voluntary licences with patent holders. The MPP works with the World Health Organization (WHO) and other partners to prioritise HIV medicines for inclusion in the patent pool, negotiates licences with key patent holders, grants non-exclusive sub-licences to multiple generic medicine manufacturers authorising them to produce and sell generic versions of patented medicines, and then works with them to ensure rapid development and availability of quality-assured medicines for use in LMICs [8,9,10]. The MPP meets with its sub-licensee on a regular basis to review progress of development, regulatory filing and approval of each product under licence. Detailed discussions are also held on challenges being faced and possible solutions suggested by MPP’s technical experts where possible, including assistance with technology transfer where applicable. MPP also facilitates API make versus buy decisions, connection with API manufacturers, shares market intelligence and analysis of WHO-MPP forecasts to help sub-licensees conduct internal prioritisation and resource allocation of projects. Sub-licensees are also provided competitive intelligence on their status of development versus other MPP licensees (identities masked) to enable them to calibrate their speed. Finally, MPP helps manufacturers prioritise countries to register products in, to ensure maximum coverage of generic products in LMICs. With a view to ensure adequate competition, the MPP also identifies and engages with new manufacturers that may have the potential to increase availability of generics and competition within the industry.

The terms and conditions of a licence can determine the degree of competition in the market and therefore the resulting equilibrium price [11], and hence the MPP licences ensure inclusion of terms and conditions intended to foster generic competition, maximise cost savings and impact on public health, including: broad geographical scope (i.e. often covering countries home to more than 90% of people living with HIV [PLHIV] in LMICs); non-exclusivity; speedy development of generic versions; ability to develop FDCs; technology transfer from patent holder to generic manufacturers; and compatibility with public health safeguards outlined in the World Trade Organization Agreement on Trade Related Aspects of Intellectual Property Rights (TRIPS) [12], including a government’s right to issue compulsory licences. MPP seeks sub-licences with multiple generic firms in order to stimulate competition and ensure adequate supply of ARVs in LMICs.

The impact of initiatives such as the MPP, however, have not been quantified. In this study, we estimate the cost savings enabled by the licences negotiated by the MPP for ARV medicines to treat HIV/AIDS in LMICs for the period 2010–2028.

## Methods

### Analytical approach

We estimated the cost savings attributable to the MPP by subtracting the price of ARV medications expected as a result of the MPP licences from a counterfactual situation in which the MPP does not exist. Cost savings were estimated between 2010 to 2028, the year by which patents on all of these medications will have expired. The existence of the MPP is assumed to impact the price of ARVs in the countries that are able to access this price. The total number of PLHIV accessing ART from 2010–2028 is based on actual numbers as reported by UNAIDS till 2015 [13] and projections of HIV coverage under the UNAIDS Fast Track scenario thereafter [3,4]. A cost-benefit ratio was calculated by comparing the actual and projected savings to the actual and projected operating expenses of the MPP over the same time period.

The calculation of ARV cost and MPP attributable savings depends on several key inputs including: ARVs licensed to MPP, the calendar years in which a particular ARV patent is in force and in which that ARV has a licence from MPP, the number of PLHIV currently or projected to be using the ARV in each country affected by MPP licences, difference between originator and generic prices, and the difference in royalties payable by generic manufacturers to patent holders with and without MPP licences. For each of these variables, we describe below how we calculated the savings related to them; we then aggregated these for total savings attributable to MPP licences. The assumptions are separately listed in Table 1 below:

**Table 1:** Key assumptions used in the model.

Assumption	Details
ARVs included	Basis MPP’s Priority Document (details in Table 2)
Timeline	2012–2028
Duration of Impact	Based on projected start—date of impact and expiry date of blocking patents
PLHIVs on treatment	Based on UNAIDS Fast track report [3,4]
ARV Market share	Based on forecasts carried out by the MPP in collaboration with the WHO from 2015 to 2025 [14]
Countries included	Low and Middle income countries as per World Bank
Number of countries impacted by licences	Impact of MPP was attributed to only those countries where the licence had unblocked an existing patent
Baseline Price	Based on originator tiered pricing as per Untangling the Web of Price Reductions by MSF
Impacted Price	Applied an international industry historic average of year-over-year erosion rates of generic product prices
Royalty saving	Basis royalty rates in agreements before and after the MPP licence
Net present value	Discounting factor of 3.5% used to obtain Net Present Value
Probability Factor	Based on the level of negotiations
Market Assumptions	No change in economic environment
MPP Operating Cost	Actual expenses and grant received till date. For years 2021–2028, projected costs are associated only with the management of medicine development projects for ARVs licensed by MPP

### Products Licensed by MPP

We included all products licensed to the MPP in the analysis. As of June 2016, the MPP had signed agreements with seven patent holders covering 13 ARVs (Table 2). MPP has sub-licensed with 12 generic manufacturers who are working on more than 60 projects based on MPP licences to develop ARV medicines [9,15].

**Table 2:** Agreements for ARV medicines with the MPP.

Licenced product	Patent Holder	Place in Treatment (as per WHO 2015 Guidelines)	Date of licence	Originator Patent Expiry Date	Number of Sub-licensees
Abacavir paed (ABC)	ViiV Healthcare	First line for pediatrics	Feb-13	2018	1
Atazanavir (ATV)	Bristol Myers Squibb	Second-line	Dec-13	2018	5
Cobicistat (COBI)	Gilead Sciences	Pharmacokinetic booster	Jul-11	2027	8
Darunavir^a^ (DRV)	National Institutes of Health	Third-line and second-line	Sep-10	2016	-
Dolutegravir (DTG)	ViiV Healthcare	First-line and third-line adults	Apr-14	2026	9
Elvitegravir (EVG)	Gilead Sciences	New ARV	Jul-11	2023	7
Emtricitabine (FTC)	Gilead Sciences	First-line and second-line	Jul-11	2024	9
Lopinavir (LPV)	AbbVie Inc	First line for paediatrics and second-line for adults and paediatrics	Nov-14 Dec-15	2026	2
Raltegravir paed (RAL)	MSD (Merck in the US and Canada)	Third-line	Feb-15	2022	2
Ritonavir (RTV)	AbbVie Inc	Pharmacokinetic booster	Nov-14 Dec-15	2026	2
Tenofovir alafenamide (TAF)	Gilead Sciences	First-line	Jul-14	2021	9
Tenofovir disoproxil fumarate (TDF)	Gilead Sciences	First-line	Jul-11	2018	3
Valganciclovir^b^	Hoffman-La Roche	Opportunistic infection	Aug-13	2016	

^a^ In September 2010, the MPP obtained a licence on darunavir-related patents from the US National Institutes of Health. At the time, however, there were other patents on DRV held by other patent holders.

^b^ Small market size did not warrant generic competition hence price agreement with option of patent licence negotiated with patent holder.

### Duration of impact

The duration of effect of MPP licences depends on when the MPP agreement with patent holder goes into effect; how long it takes for MPP to sub-licence to generic manufactures; how long it takes for generic manufactures to bring product to market; and how long a blocking patent will be in effect [16].

To determine when the impact of MPP began, we assumed that for ARVs with existing generic versions available on the market, the impact of the licence starts in the year an agreement by the patent holder with MPP is reached. Based on actual experience, we assumed that generic manufacturers would sign sub-licences immediately after MPP obtains a licence from the patent holder.

For ARVs where generic versions are yet not available, a 1–3 year lag was assumed from the time of an MPP agreement with the patent holder to the actual market entry of a generic to allow for a generic manufacturer to develop product and obtain regulatory approval. The choice of a 1, 2 or 3 year time-lag depended on the time required for development of each specific product.

For MPP licences on pipeline ARVs (those for which the patent holder had not obtained regulatory approval, or obtained one recently), we assumed a lag of 3–5 years after regulatory approval of originator drug before generic versions would be available in the market. We made the assumption that impact of MPP licences would last one year after the originator’s patent expires. This is due to the fact that there is some lag, usually at least 1 year, before multiple generic products enter the market after patent expiry (this being the time taken for national regulatory approvals in multiple countries).

The time taken for development of paediatric products is different from adult formulations, since clinical data for paediatric formulations generally take longer to be available. Hence, we have separately calculated the impact period for adult and paediatric formulations.

The time period of impact for each ARV is listed below in Table 3 and Table 4. Shading indicates start and finish years for impact.

**Table 3:** Impact timelines by adult ARV treatment.

	Years																		
ARV	20	20	20	20	20	20	20	20	20	20	20	20	20	20	20	20	20	20	20
	10	11	12	13	14	15	16	17	18	19	20	21	22	23	24	25	26	27	28
**ATV**																			
**DRV**																			
**LPV/r**																			
**RAL**																			
**RTV**																			
**TDF**																			
**TDF/FTC**																			
**COBI**																			
**EVG**																			
**DTG**																			
**TAF**																			

Shading indicates period of impact.

**Table 4:** Impact timelines by paediatric ARV treatment.

	Years																		
ARV	20	20	20	20	20	20	20	20	20	20	20	20	20	20	20	20	20	20	20
	10	11	12	13	14	15	16	17	18	19	20	21	22	23	24	25	26	27	28
**ABC**																			
**ATV**																			
**DRV**																			
**LPV/r**																			
**RAL**																			
**RTV**																			
**TDF**																			
**TDF/FTC**																			
**COBI**																			
**EVG**																			
**DTG**																			
**TAF**																			

Shading indicates period of impact.

### PLHIV on treatment

**Fig 1:**
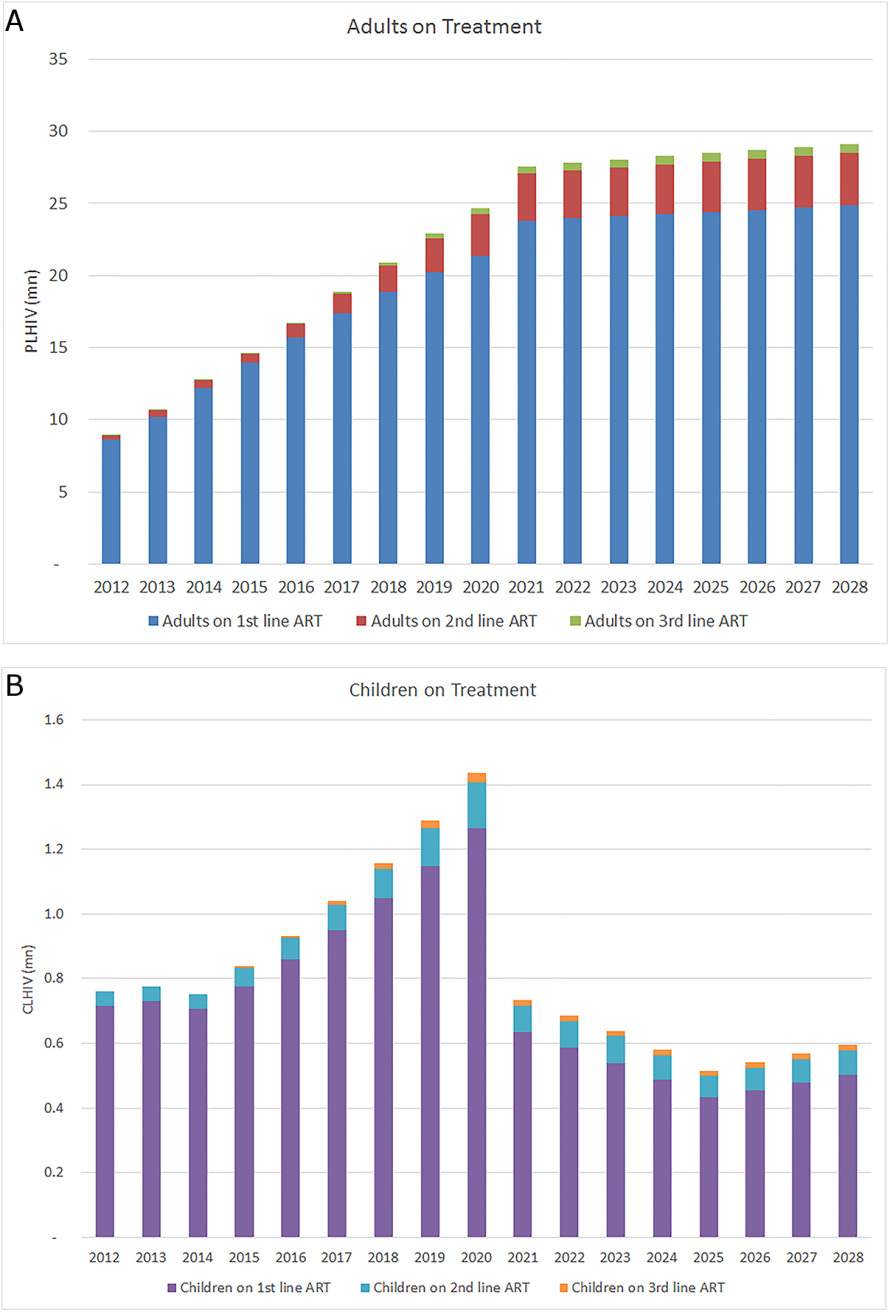
Aggregate adults (Fig 1A) living with HIV and children (Fig 1B) living with HIV on treatment by year, regimen line, and age-group.

The model is based on actual savings generated by MPP licences up until 2015 [17] and projected estimates of PLHIV accessing ART in LMICs in future, drawn from UNAIDS Fast Track report [3,4,18]. This scenario anticipates aggressive scale-up to 90% of PLHIV identified, and 90% of the identified PLHIV accessing treatment regardless of CD4 level, aiming to ensure that 30 million people living with HIV have access to treatment by 2020. The strategy also includes rapid scale up of treatment to reduce mother to child transmission of HIV, which is shown in Fig 1 through the rapid reduction of children on treatment post 2020. Our model uses the forecasted proportion of PLHIV on first, second, and third (salvage) line treatment regimens in each year. Based on the UNAIDS report, the numbers of PLHIV on treatment by regimen line and age-group are shown in Fig 1.

### ARV market share

We estimated the number of potentially impacted PLHIV for a particular ARV in a given year based on the drug’s market share in that year. Market share was projected for each ARV, separately for adults and paediatrics, and by regimen line, based on forecasts carried out by the MPP in collaboration with the WHO from 2015 to 2025 [14]. The projections take into account the WHO 2015 Guidelines [19] for existing products and also forecast a likely uptake of pipeline products (those that have not yet received regulatory approval). Market share of each individual drug and fixed-dose combinations (FDC) was calculated separately based on forecasted use. We assumed that the presence of MPP would not affect market share. For the years 2026 to 2028, we have assumed constant market share for each drug.

### Number of countries impacted by licences

A patent-holder in a country has sole rights to commercialise a product in that country for a fixed period of time. In order for a generic medicine to reach a patient, there must not be patents blocking the production of that medicine in the exporting country nor patents blocking entry of the generic in the importing country–in other words, a clear legal pathway is required in both the exporting and importing country. For each ARV licensed by the MPP, we identified patent status in both exporting and importing countries. We attributed impact to the MPP only in those countries where the licence had unblocked an existing patent.

In other words, for each ARV in each country, we considered MPP to have had an impact only if all three of the following conditions were met: (1) the importing country had a blocking patent for that ARV [16]; (2) the importing country was not included in a prior voluntary licence for the ARV [20–24]; and (3) the importing country was included in the MPP licence for that ARV [25].

In addition, we have made the assumption that each originator’s existing voluntary licensing programme will be applied to all their future HIV medicines, with the same geographical scope, hence the pre-MPP geographical scope of originators is not counted in the MPP impact. Only incremental territory negotiated by MPP is considered for MPP’s impact. Example, ViiV’s Territory as per their voluntary licence was 67 countries [24], which increased to 92 countries post MPP intervention [26]. Under the MPP licence, generics manufacturers are also allowed to sell in any country where they do not infringe any patent, leading to access in a total of 131 countries. MPP impact is attributed only to those additional 64 countries (131–67 = 64).

We then considered patent status in the exporting country of manufacture. India is the key exporting country, presently supplying more than 95% of generic ARVs to LMICs [27,28]. Therefore, if there was a blocking patent in India on which MPP obtained a licence, we attributed impact in every country incrementally included in the licence, (unless a country was already included in a bilateral license from the patent holder).

For some ARVs in some countries, patent information was not available; in such cases, we made the conservative assumption of no impact by the MPP. In cases where MPP has not yet concluded a patent licence, geographical scope was assumed to be the same as for MPP’s first licence on tenofovir (TDF) with Gilead covering 112 countries.

### ARV price

The price of generic drugs typically decreases as volumes and competition increases, which means that the price gap between generic and originator versions of medicines widens over time and with the entry of new players. In our model, the monetary value of MPP’s work is driven by the gap between the originator’s baseline price for an ARV and the lower price resulting from generic competition enabled by MPP.

The baseline price is the originator’s tiered price for LMICs, as quoted in the Médecins Sans Frontières, *Untangling the Web of Price Reductions* annual report [29]. Where originators have more than one price tier, a weighted average was used. The originators’ tiered prices were projected to decline at a rate based on price movements over the previous three-year period [30,31]. Specifically in case of LPV/r, where originator price is lower than generics price, the originator tiered price is used as a baseline price.

For pipeline ARVs for which tiered prices were not yet available, the originator’s baseline tiered price was estimated by applying the ratio of the ARV–tenofovir disoproxil fumarate–weighted average tiered price in LMICs to its price in the USA. TDF was used as a proxy in this case because it is the most recent originator ARV to be widely launched in developed and developing countries and we assume it is reflective of originator pharmaceutical companies’ current approach to ARV pricing in developed versus developing countries.

**Fig 2:**
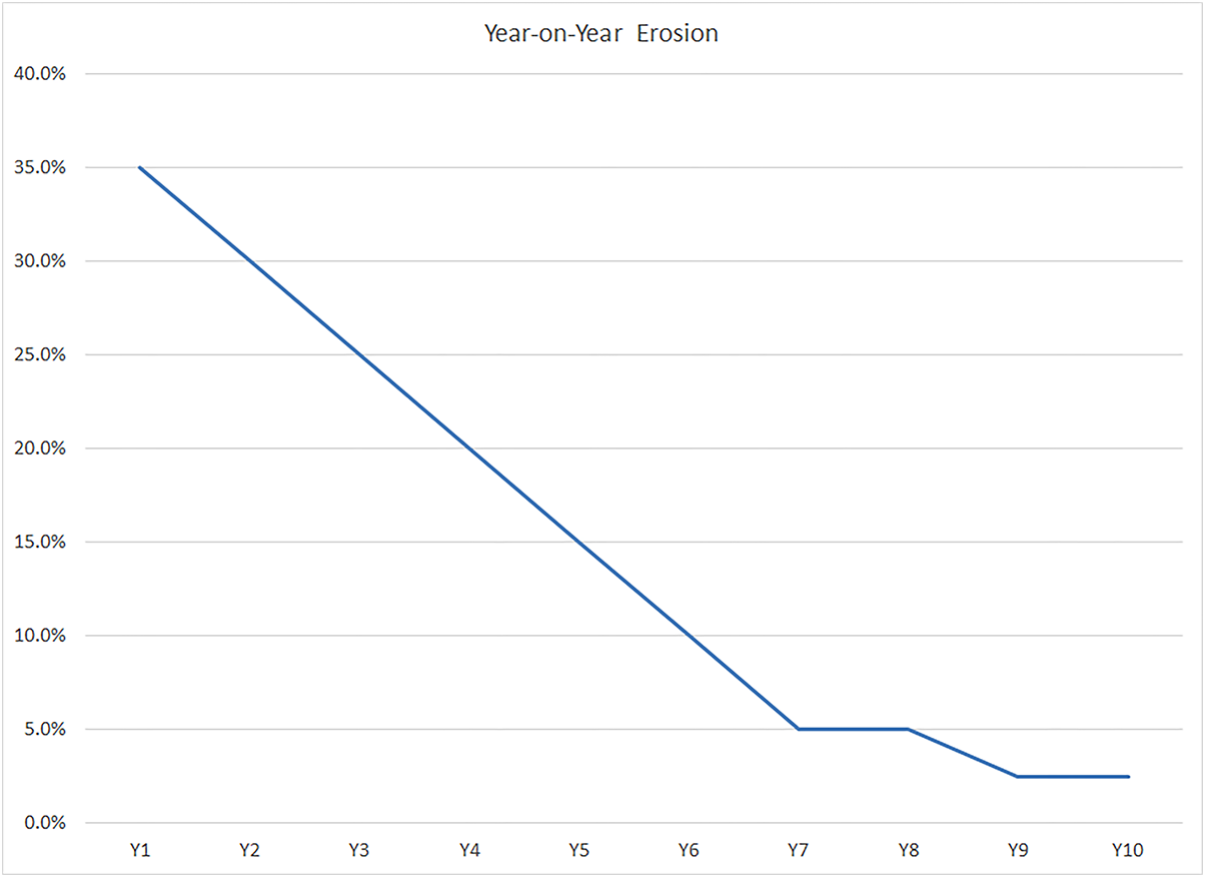
Generic price erosion (small molecule)–international markets [32]. The erosion is reported as a percentage of the brand name version price in that year.

To obtain the future projected generic prices for existing ARVs, we used an international industry historic average of year-over-year erosion rates of generic product prices. The reduced price schedule begins at the impact start date for each ARV, as explained above under the duration variable. This is shown in Fig 2. Historically, it has been seen that the launch of five or more generic versions of a product triggers such price competition, and our model uses this price reduction curve only in such cases, where there are 5 or more generics in the market. For products with fewer than five competitors in the market, in all cases except one we made the conservative assumption of no price erosion.

For pipeline ARVs, where generic prices are not available, the generic launch price of the pipeline ARVs in LMICs was calculated as 2% of originator’s price in the USA, declining by 50% within 5 years and thereafter remaining constant. This is based on the usual trend in the generic industry in highly regulated and competitive markets: generics are rapidly available in LMICs for ~98% discount to originator with robust competition (five-six players) and the discount increases to ~99% in the following years [29,33].

Price erosion is significant in for-profit, high price markets where a large number of generics begin competing for the market when the period of exclusivity ends. We have assumed that a) the same intensely competitive conditions apply in each LMIC ARV market and b) the same erosion rate is applicable even if LMIC ARV markets start off with a lower base price. MPP facilitates such generic price competition by engaging with multiple generic sub-licensees for each licensed ARV and fostering early development of needed generic formulations so that multiple manufacturers come to market almost simultaneously.

### Royalty saving

In some cases, an MPP licence can decrease the level of royalty payments that were due from generic to originator companies under previous agreements. For example, before the MPP licence, some generics manufacturers had received a voluntary licence from Gilead for 95 countries, through which they had to pay 5% royalty to Gilead for sales on TDF. MPP negotiations resulted in a reduction of this royalty rate to 3% for those manufacturers who continued with a bilateral licence with Gilead. Furthermore, following MPP’s agreement with Gilead, generics manufacturers who switched to MPP licences did not have to pay any royalty if they utilised the unique flexibility of terminating TDF part of the agreement. Most generic companies that switched to the MPP licence used this flexibility [34].

We assumed that generic manufacturers will pass on 50% of any royalty savings into reduced prices for TDF. This is a conservative assumption as the market for TDF is very competitive and manufacturers are aggressively competing for market share by reducing prices. As an example of competitive pricing, note that since MPP’s first agreement with Gilead, prices of TDF have fallen from USD 223 per person per year in 2011 to USD 56 per person per year in 2015. Thus, we made the reasonable assumption that at least half of the royalty saved by MPP licensees on TDF is passed on to the buyer.

### Net present value

Since monetary savings due to the MPP and MPP’s costs are spread over several years in the future, we have applied a discount rate of 3.5% (UK treasury rate) to savings as well as costs in our model. This provides a net present value to the savings and cost-benefit ratio.

### MPP operating cost

For 2010–2015, costs were linked to MPP’s actual operating expenses of USD 22.9 million. For the period 2016–2020, the cost of USD 29.2 million is the grant received by MPP from UNITAID for continuing operations in HIV/AIDS until year 2020. For years 2021–2028, projected costs are associated only with the management of medicine development projects for ARVs licensed by the MPP and their national registrations in LMICs.

### Calculation equation

$$\begin{array}{l} \text{MPP}\;\text{Savings}=\left({\text{Savings}\;\text{of}\;\text{product}}_{1}{*\;\text{VL}\;\text{Likelihood}\;\text{of}\;\text{product}}_{1}\right)+\left({\text{Savings}\;\text{of}\;\text{product}}_{2}{*\;\text{VL}\;\text{Likelihood}\;\text{of}\;\text{product}}_{2}\right)+\ldots ..+\left({\text{Savings}\;\text{of}\;\text{product}}_{\text{n}}{*\;\text{VL}\;\text{Likelihood}\;\text{of}\;\text{product}}_{\text{n}}\right)\\ \text{Savings}\;\text{of}\;\text{Product}\;=\text{duration}\;\text{of}\;\text{use}\;\text{under}\;\text{licence}\;*\;\text{number}\;\text{of}\;\text{user}\;\text{PLHIV}\;*\;\text{price}\;\text{reduction}\\ \text{Duration}\;\text{of}\;\text{use}=\left(\text{date}\;\text{of}\;\text{patent}\;\text{expiry}\;+\;1\right)–\text{date}\;\text{of}\;\text{launch}\;\text{of}\;\text{generic}\;\text{product}\\ {\text{Number}\;\text{of}\;\text{users}=\text{PLHIV}\;\text{on}\;\text{ART}\;\text{in}\;\text{countries}\;\text{impacted}\;\text{through}\;\text{licence}\;*\;\text{market}\;\text{share}\;\text{of}\;\text{product}}_{1}\\ \text{Price}\;\text{reduction}\;=\;\text{originator}\;\text{tiered}\;\text{price}–\text{generic}\;\text{price}\;\left(\text{with}\;\text{projected}\;\text{trends}\right)\\ \begin{array}{l} \text{VL}\;\text{Likelihood}=\text{probability}\;\text{factor}\;\left(\text{explained}\;\text{in}\;\text{limitation}\;\text{section}\right)\\ \text{Where}\;\text{n}=\text{total}\;\text{number}\;\text{of}\;\text{products} \end{array} \end{array}$$

### Other assumptions

Our model is dependent on a number of key assumptions upon which the MPP has very limited or no direct influence, such as those relating to treatment financing, pharmaceutical industry practices such as those on pricing, HIV/AIDS epidemiology, WHO and individual country treatment guidelines, and regulatory approvals. The model assumes that all these factors and practices remain constant and consistent over the time period under consideration.

It should be noted, that 99% of MPP’s projected direct savings are attributable to executed licences and the remaining 1% comes from the one remaining licence of raltegravir adult formulations, for which Merck is not currently in negotiations with the MPP [this product is used as a third line or salvage option in LMICs, resulting in its low usage]. Whether each product will ultimately be licensed by MPP and contribute to the savings as per MPP approach is unknown, and we based our study on the current level of negotiations with each company. In our model, we have allocated a probability factor for each product on the basis of this level of negotiation. For example, for medicines where MPP negotiations are complete and a licence has been signed, the probability factor is 100% and all the savings are taken in account in the model. On the other hand, in cases where companies are not yet in negotiations with MPP we conservatively assumed only 25% of the savings will be attributed to these products in the final savings value, since there is a lower chance an agreement will ultimately be reached. Similarly, 50% of the savings are included for products where negotiations have just started and 75% for those in late stages of negotiations. This assumption was based on MPP team’s collective judgment on current status of negotiations and experience with specific patent holding companies.

The model also assumed that WHO guidance for ART will remain consistent over the coming years. However, the WHO’s 2016 guidance of Test and Treat [19] has raised the number of people needing treatment from 28 million to 37 million worldwide. Other significant changes in the guidelines could have a strong impact on this analysis, including a potential recommendation to include integrase inhibitors (INIs) in first-line treatment resulting in a dramatic uptake in market adoption of INIs. This could lead to an increase in economic savings attributable to the MPP, given that key INIs have been licensed to the MPP and are also widely patented.

## Results

### Savings

**Fig 3:**
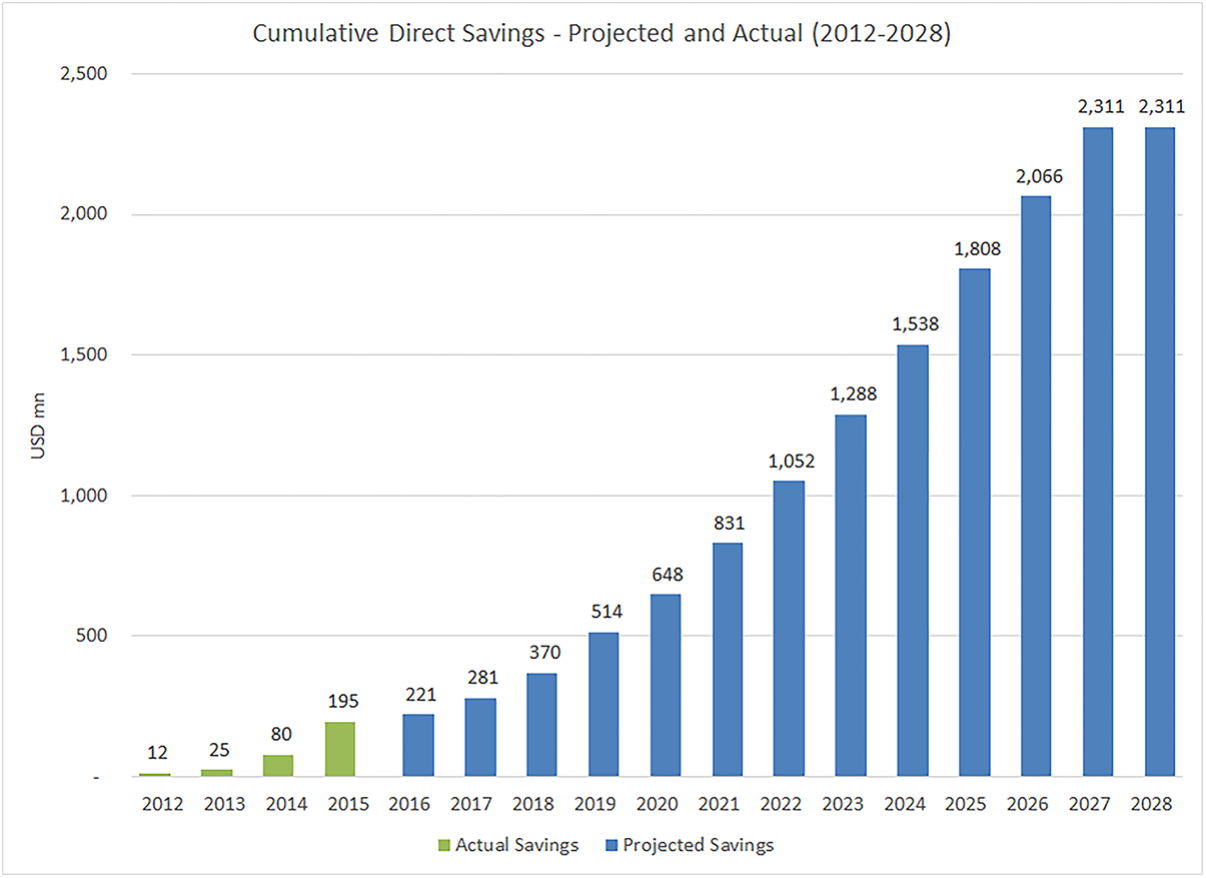
Cumulative direct economic global savings in LMICs by year.

This analysis estimates the MPP’s voluntary licensing activities could yield USD 2.3 billion in savings from 2010–2028, representing an estimated cost-benefit ratio of 1:43. Fig 3 shows MPP’s estimated actual savings from 2012–2015 [17], as well as projected savings (2016–2028). We report that actual savings from generic competition enabled by MPP began in 2012, primarily on account of royalty savings by generic manufacturers through MPP negotiated agreements, and initially to a lesser degree, increased competition for certain ARV formulations in a number of countries, made possible through MPP agreements.

Between 2015 and 2018, we found that MPP licences will result in increasing access to generic medicines in countries with patent barriers, peaking in 2019, after which the patents for a number of targeted medicines will expire (at which time the MPP impact for licences on those products will end). We report that growth rate of savings will likely begin increasing again in 2021 with the uptake of newer ARVs licensed by MPP (e.g. DTG) and continue to rise until 2027. Between 2027 and 2028, our data show that there will be almost no additional savings, because patents on all the newer ARVs licensed by MPP will expire.

### Impacted patients

**Fig 4:**
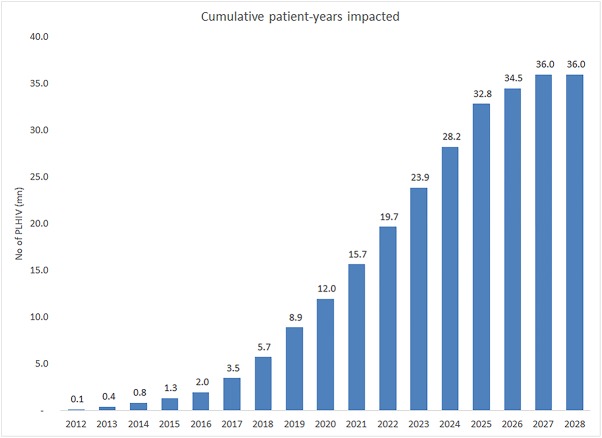
Cumulative patient-years impacted by MPP licence agreements.

The total number of patient-years impacted by MPP’s agreements is shown in Fig 4. In 2025, more than 95% of all PLHIV on treatment are expected to be on regimens whose price is impacted by MPP deals, an increase from 75% in 2013. Of these PLHIV on treatment, about 15.9% are unlikely to have received these regimens if MPP deals were not in place (calculated as the number of PLHIV on treatment in countries where valid patents would have blocked generic entry if they had not been included in MPP licences). This analysis predicts the MPP’s voluntary licensing activities could result in a cumulative 36 million patient-years getting lower cost treatment by the year 2027.

The saving of USD 2.3 billion is equivalent to more than 24 million PLHIV receiving first-line ART in LMICs for 1 year at average prices today [28].

### Cost-benefit ratio

The cost-benefit ratio is calculated as direct savings generated by MPP’s licensing agreements. Based on the MPP model, the cost-benefit ratio is projected to be 1:43, meaning the total direct savings calculated at the end of 2028 will be about 43 times the funds used in executing the primary mission of the MPP (Table 5).

**Table 5:** Total direct savings and costs by time period (USD).

	Total: 2012–28
**Savings**	USD 2,311.0 m
**Costs**	USD 53.8 m
**Cost-benefit ratio**	1:43

## Discussion

The MPP approach has the potential to generate large savings and deliver high cost-benefit ratios with current estimates and forecasts showing time-discounted savings. This study shows savings may be as high as USD 2.3 billion between 2010–2028 over a cost base of a little over USD 50 million over this 18-year time-frame. The MPP licences can produce large-scale cost-savings for national treatment programmes in LMICs, and result in 36 million cumulative patients-years getting lower cost treatment.

Our model calculates the costs of ARVs for a certain number of people in a particular year, with savings generated by the difference between the generic price enabled by the MPP versus the tiered prices originators offer in LMICs. The model assumes the number of people treated will be the same irrespective of the existence of the MPP. However, in reality, if drug prices were significantly higher (e.g., in absence of MPP), it is likely that there would be fewer people on treatment since budget envelopes are relatively static. Hence the result of MPP’s existence will likely be a mix of cost savings and life-years saved. However, we have focused only on financial savings in our analysis and not lives saved.

As our model is dependent on a number of assumptions, a change in such assumptions would affect the overall savings calculated through the model. We have done extensive sensitivity analysis on the effect of such changes on savings. Results of some key sensitivity calculations are presented here: (i) in case there is no further licensing to what is already executed, the minimum savings expected from MPP licences would be USD 2.29 billion–from those licenses which have already been executed and out-licensed to generic manufacturers; (ii) impact of delay in availability of products and hence a delay in start of impact realisation, which could be due to: delay in licence execution with originator, delay in generic ramp-up of manufacturing and selling, or delay in market uptake. For every year of such delay, roughly USD 100 million to USD 200 million is forfeited from being realised in savings; (iii) impact due to early availability of drugs: similarly, there could be an increase in impact period due to early market entry or early uptake of products, resulting in an increase of impact by USD 100 million to USD 200 million (depending on the market share of such product) for every such year; (iv) a difference in savings could be realised should originators and/or generic manufacturers adjust their pricing practices. If the model’s pricing gap projections are higher or lower by a net 20%, the impact on total savings would be USD 462 million and (v) in case a discounting factor is not used for calculation of the net present value of savings, the expected savings increase to USD 2.98 billion, resulting in a cost-benefit ratio of 1:51.

Additionally, there are other benefits of MPP activities that the study does not include. For example, its Patent Status Database [16]–the largest repository of information on HIV patents in developing countries–informs procurement decisions of many stakeholders such as governments and international donors, and may facilitate purchase of lower-priced generics by providing increased legal certainty to do so. The publication of the full text of MPP licences has also established a precedent of increased transparency in this field.

This analysis shows that it is possible to estimate the impact of global policy initiatives intending to reduce the price and improve access to medicines. Other studies examining similar initiatives are needed to better inform decision-making. Furthermore, this study demonstrates the potential cost-savings and public health benefit generated through widespread availability of generic medicines via licensing in LMICs, with potential application to other therapeutic areas beyond HIV. In 2015 the MPP expanded the scope of its work to include tuberculosis and hepatitis C. GlaxoSmithKline has also offered to license patents on its cancer medicines to the MPP for use in developing countries [35]. Licences authorising generic versions of widely patented medicines in a broad range of LMICs have the potential to save billions of dollars and impact on millions of lives.
